# Monogenic and multifactorial autoinflammatory diseases: Clinical and laboratory characterization in a pediatric Saudi population

**DOI:** 10.1186/1546-0096-13-S1-P160

**Published:** 2015-09-28

**Authors:** S Al-Mayouf, A Alsonbul

**Affiliations:** 1 King Faisal Specialist Hospital & Research Centre, Pediatric Rheumatology, Riyadh, Saudi Arabia

## Objective

To report on the clinical and laboratory features of both monogenic and multifactorial autoinflammatory diseases in Saudi children.

## Methods

This retrospective report comprised all children with autoinflammatory diseases treated at the Pediatric Rheumatology Clinic at King Faisal Specialist Hospital and Research Center, Riyadh, between January 2000 and December 2014. Demographic characteristics, diagnosis, age at onset, disease duration, follow-up duration, clinical features and laboratory variables including genetic results if available, and treatment were collected.

## Results

A total of 75 patients (43 females) with various autoinflammatory diseases were included; consanguinity was present in 45%. The mean age was 11.6 years with mean age at onset of 2.7 years and mean disease duration was 8 years. Patients were diagnosed as follows: familial Mediterranean fever (FMF) 19; chronic recurrent multifocal osteomyelitis (CRMO) 18; monogenic form of systemic onset juvenile idiopathic arthritis (So-JIA) 14, early onset sarcoidosis 7, familial Behect's disease 5, periodic fever, aphthosis, pharyngitis and adenitis (PFAPA) 4, CINCA 3. Five patients had periodic fever but without definite diagnosis. Most of the cases were referred with inaccurate diagnosis. FMF patients had the usual manifestations but one patient had sacroiliitis. *MEFV* genetic testing showed pathogenic mutations of *M694V* gene in 12 patients while 7 patients had heterogonous sequence variants. All FMF patients had favorable response to colchicine. All CRMO patients presented with bone pain and fever with elevated inflammatory markers and abnormal radiographic findings. Biopsy results were consistent with osteomyelitis, but cultures were negative. All CRMO patients had favorable response to treatment (16 treated with pamidronate and 3 patients required infliximab). Patients with So-JIA had autosomal-recessive pattern on inheritance and whole-exome sequencing identified a homoallelic missense mutation in LACC1. Patients with early onset sarcoidosis had multi-organ involvement, diagnosis was proven by histopathology with negative cultures and treatment included prednisone, methotrexate and biologic agents. Three PFAPA patients responded well to corticosteroid and one patient underwent tonsillectomy. All CINCA patients had a good response to IL-1 blocker.

## Conclusion

Autoinflammatory diseases other than FMF may be overlooked in our region. Increased awareness among pediatricians is needed for timely and accurate diagnosis and proper management. Association of LACC1 with monogenic So-JIA justifies investigation of its role in autoinflammatory disorders.

